# Barriers to Healthcare Access and Utilization Among Immigrants in Host Countries: A Systematic Review of Qualitative and Quantitative Evidence

**DOI:** 10.7759/cureus.104278

**Published:** 2026-02-26

**Authors:** Ferid Krupić, Melissa Krupić, Saidarab Sahra, Emina Dervišević, Nail Seffo, Jasmin Alić

**Affiliations:** 1 Department of Anaesthesiology, Institute of Clinical Sciences, Sahlgrenska Academy, University of Gothenburg, Gothenburg, SWE; 2 Department of Gynaecology, Sahlgrenska University Hospital/Östra, Gothenburg, SWE; 3 Department of Maternity Care, Sahlgrenska University Hospital, Gothenburg, SWE; 4 Institute of Health and Care Sciences, Sahlgrenska Academy, University of Gothenburg, Gothenburg, SWE; 5 Department of Forensic Medicine, University of Sarajevo, Faculty of Medicine, Sarajevo, BIH; 6 Urology Clinic, Clinical Center University of Sarajevo, Sarajevo, BIH

**Keywords:** access to healthcare, cultural competency, emigrants and immigrants, healthcare access disparity, health services accessibility, language barrier

## Abstract

Immigrant populations frequently encounter barriers when accessing healthcare services, potentially affecting patient safety, healthcare utilization, and clinical outcomes. Understanding these barriers is essential for improving equitable and patient-centered care.

A systematic review of qualitative and quantitative studies was conducted in accordance with PRISMA 2020 guidelines. PubMed/MedLINE, Embase, Cochrane Library, PsycINFO, EconLit, Web of Science (WoS), and CINAHL were searched from January 2005 to August 2023. Inductive thematic analysis was used to synthesize findings across studies. The review was not prospectively registered, included only English-language studies, and relied predominantly on qualitative evidence. Heterogeneity across study designs and healthcare settings may limit generalizability. The authors received no external funding for this study.

Three interconnected themes consistently emerged: limited transcultural competence, language barriers, and discrimination in healthcare. Inadequate cultural competence was associated with communication difficulties and reduced care effectiveness. Language barriers contributed to miscommunication, delayed care, and increased healthcare utilization. Experiences of discrimination were linked to reduced trust in healthcare systems and poorer patient engagement. These factors negatively influenced patient safety, satisfaction, and clinical outcomes.

Immigrant patients face persistent and interrelated barriers to healthcare access. Strengthening culturally responsive care, improving access to professional interpreter services, and addressing discriminatory practices are essential to improving patient safety, satisfaction, and clinical outcomes. Future research should evaluate targeted interventions aimed at improving communication, cultural competence, and healthcare equity.

## Introduction and background

Migration occurs for multiple reasons, including security concerns, demographic pressures, human rights issues, poverty, and climate change. According to Eurostat, as of January 1, 2021, approximately 23.7 million third-country nationals were residing in the European Union (EU), representing 5.3% of the total EU population [1]. Migration may be voluntary or forced and is commonly driven by economic, environmental, and social factors. These movements are influenced by push factors, which compel individuals to leave their country of origin, and pull factors, which attract them to a specific destination country [2]. Broadly, migration is motivated by social and political circumstances, demographic and economic conditions, and environmental or climate-related changes [1,2].

In addition to economic and voluntary migration, armed conflicts and political instability remain major drivers of global displacement. Individuals forced to migrate due to war often face increased health vulnerability, including trauma, disrupted continuity of care, language barriers, and limited familiarity with healthcare systems in host countries [3]. These factors may contribute to delayed healthcare access, unmet medical needs, and poorer health outcomes. Recognizing the healthcare challenges faced by conflict-affected migrants is important for developing equitable and responsive healthcare services [3,4].

Following migration, individuals retain their legal rights and responsibilities in the host country. One fundamental right, irrespective of country of origin, is access to healthcare and medical services. Increasing global migration has contributed to growing multiculturalism, placing new and complex demands on healthcare systems [1,2]. Encounters between healthcare professionals and patients with an immigrant background often involve specific challenges, and ensuring equitable, high-quality care remains a core professional responsibility [4]. Achieving this goal requires culturally responsive healthcare environments and healthcare professionals with adequate transcultural competence [5].

Previous research has consistently reported barriers and difficulties faced by immigrants when accessing healthcare services. A key contributor to health disparities between immigrant and native populations is delayed healthcare-seeking behavior among immigrants, resulting in later utilization of health services compared with native residents [6-8]. These differences raise important questions regarding the underlying causes of delayed care-seeking. Documented contributing factors include ethnicity, socioeconomic status, and cultural influences on healthcare interactions [9-11]. Additional factors such as immigrant status, marital status, age, gender, number of children, income, and educational level further influence healthcare utilization [12]. Language barriers, including limited proficiency in the host-country language and challenges related to interpreter services, have also been widely reported [13-15]. In some cases, delayed or inadequate use of healthcare services has been associated with increased mortality among immigrant populations [16,17]. Transcultural competence among healthcare professionals was identified as an important factor influencing the quality of care for immigrant patients [18-27].

Despite growing recognition of healthcare disparities among immigrant populations, existing research remains fragmented across qualitative and quantitative domains, often focusing on isolated determinants such as communication barriers [4,5,8-10], cultural differences [26,28-40], or discrimination [41-47]. Few studies have comprehensively synthesized these interrelated factors across diverse healthcare systems and settings, limiting the ability to draw broader conclusions about systemic barriers affecting immigrant patients [6,7,11].

The objective of this systematic review was to identify and synthesize available qualitative and quantitative evidence on the barriers faced by immigrant patients in accessing and receiving healthcare in host countries. Specifically, this review aimed to examine how cultural competence, language barriers, and discrimination influence healthcare experiences, access, and outcomes among immigrant populations.

The review was guided by the following research question: Among immigrant populations, what barriers influence access to and utilization of healthcare services in host countries, and how do these barriers affect patient experiences and healthcare outcomes?

## Review

Methods

This systematic review was conducted in accordance with the PRISMA 2020 (Preferred Reporting Items for Systematic Reviews and Meta-Analyses) guidelines [18]. Given the aim of the study to explore factors influencing individuals’ experiences and decision-making processes, qualitative studies were considered particularly suitable. Qualitative methodology enables in-depth exploration of lived experiences and perceptions, which aligns with the objective of identifying factors that shape healthcare-related decisions among individuals [19].

Selection and Eligibility Criteria

The initial selection strategy was developed in consultation with an information specialist at the University Library of Gothenburg. Following this consultation, two authors independently conducted the literature search and study selection. 

Studies were considered eligible if they investigated immigrants’ or migrants’ experiences in accessing or utilizing healthcare services. We included qualitative, quantitative, and mixed-methods studies published in peer-reviewed journals, available in full text, and written in English within the predefined time frame (between January 2005 and August 2023). English was used due to resource constraints. The date range reflects the modern era of increased global migration and evolving healthcare policies. Mixed-methods studies and systematic reviews were excluded. Studies involving adult populations (≥18 years) were eligible. To be included, studies had to address at least one of the following domains: transcultural competence, communication and language barriers, discrimination in healthcare, access to healthcare services, or quality of care.

We excluded review articles, editorials, commentaries, case reports, and conference abstracts without full text. Studies that did not distinguish immigrant populations from the general population, as well as those lacking sufficient methodological detail, were excluded. Non-English publications, duplicate records, and gray literature were also excluded. Additionally, studies focusing exclusively on epidemiological data without examining patient experience or interaction with healthcare systems were not considered eligible.

In accordance with a PICO-informed framework, the population of interest included immigrant patients, the exposure involved healthcare access and utilization, comparisons (where applicable) included native populations or differing healthcare contexts, and outcomes included patient experiences, barriers, and healthcare-related outcomes.

Literature Search Strategy

Search terms were developed using SweMeSH to enhance precision and relevance. The primary keywords, followed by secondary in parenthesis, included "healthcare access disparities" ("health services accessibility", "healthcare access", "health care utilization", "access to healthcare", "barriers to healthcare", "healthcare disparities"), "immigrants" ("migrants", "emigrants", "refugees"), "language barriers" ("communication barriers", "language barriers", "interpreter services", "health communication"), "cultural competency" ("cultural competence", "transcultural care", "cross-cultural care", "culturally competent care"), "discrimination" ("healthcare discrimination", "inequality", "health inequities"). These terms were combined using the Boolean operator AND, while the operator OR was used for the inclusion of secondary keywords. MeSH terms and free-text keywords related to community, healthcare access, and influencing factors were integrated into the search strategy. Searches were conducted in PubMed/MedLINE, Cocrane Library, CINAHL, Embase, Web of Science (WoS), PsycINFO, and EconLit. In addition, reference lists of relevant studies and reviews were manually screened (Table 1).

**Table 1:** Results of database searches

Database	Hits
PubMed/MedLINE	255
Cocrane Library	134
Embase	98
Others	64
Total	551

Titles of all identified studies were initially reviewed, followed by abstract screening. Articles whose abstracts were deemed relevant to the study aim were retrieved in full text. Of the 45 full-text articles assessed, 15 studies met the inclusion criteria and were included in the final analysis.

Data Analysis

All authors independently screened abstracts and full-text articles for eligibility. The included studies were analyzed using a five-step thematic analysis model [21]. First, all authors read the selected studies with particular emphasis on the Results section. Key findings were then identified, after which similarities and differences across studies were compared. Data extraction and organization were performed using Microsoft Excel (Microsoft Corporation, Redmond, WA, USA). The software was used to structure extracted variables, perform descriptive data handling, and generate summary tables. These comparisons informed the development of new themes and subthemes. In the final step, an integrated synthesis was constructed, with the identified themes and subthemes forming the basis for the Results section. Discrepancies were discussed among all authors until consensus was reached.

Risk-of-Bias Assessment

The methodological quality and risk of bias of the included studies were assessed using validated tools appropriate to the study design. Qualitative studies were evaluated using the Critical Appraisal Skills Programme (CASP) Qualitative Checklist, while observational quantitative studies were assessed using the Risk Of Bias In Non-randomized Studies of Interventions (ROBINS-I) tool.

Each study was independently appraised across relevant domains, and an overall judgment was assigned. This structured approach enabled transparent evaluation of methodological quality across heterogeneous study designs and supported interpretation of the findings.

Ethical Considerations

Ethical aspects of all included studies were carefully reviewed. All studies adhered to established research ethics guidelines, including informed consent obtained verbally and in writing from participants, voluntary participation, confidentiality of data, and approval from appropriate ethics committees. Participants in the original studies were informed of their right to withdraw at any time without consequences, and all collected data were used solely for research purposes.

Results

Literature Search and Study Selection

The study selection process was conducted in accordance with PRISMA 2020 guidelines [18]. Database searches identified a total of 551 records, with no additional records identified through manual searching of reference lists and other sources. After removal of 326 duplicates, 225 records remained for title and abstract screening.

Following initial screening, 180 articles were excluded for not meeting the inclusion criteria. The full texts of 45 potentially eligible studies were retrieved and assessed for eligibility. Of these, 30 studies were excluded due to reasons such as irrelevance to the study objective, insufficient methodological quality, lack of focus on immigrant populations, or absence of relevant outcomes.

**Figure 1:**
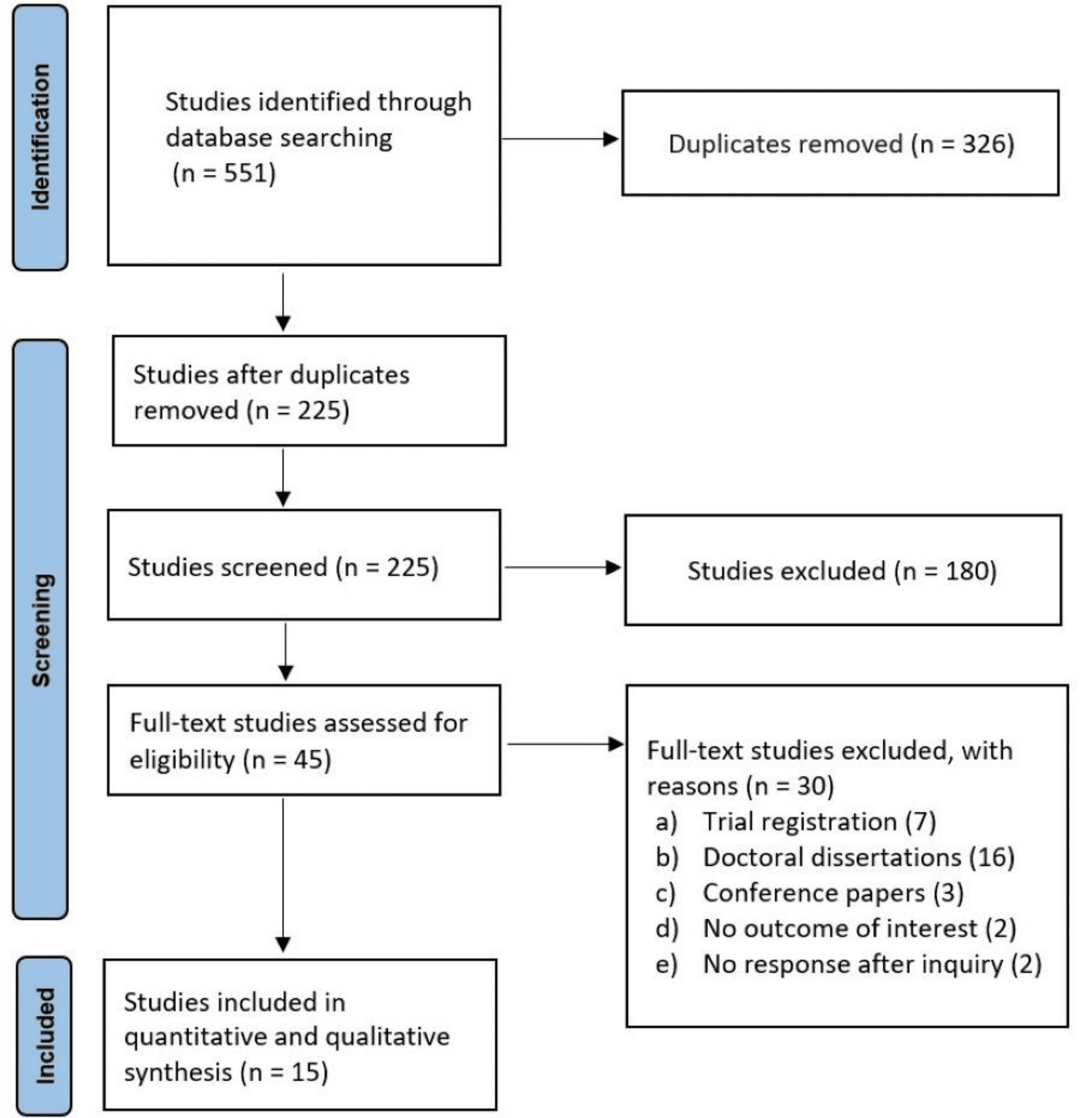
PRISMA guidelines PRISMA, Preferred Reporting Items for Systematic Reviews and Meta-Analyses.

Ultimately, 15 studies met the inclusion criteria and were included in the final qualitative and quantitative synthesis. The detailed study selection process is presented in the PRISMA flow diagram (Figure 1). Publications spanned from 2005 to 2023, with data predominantly collected through semi-structured interviews and focus group discussions. 

A total of 15 studies were included in the final synthesis, comprising both qualitative and quantitative designs (Table 2). The majority of included studies were qualitative, primarily using semi-structured interviews to explore experiences of immigrant patients and healthcare professionals, while two studies employed quantitative survey methodologies [4,37]. Sample sizes varied across studies, reflecting differences in study design and methodological approach.

**Table 2:** Characteristics, limitations, thematic domain, and risk of bias of included studies NR = not reported; CASP = Critical Appraisal Skills Programme, ROBINS-I = Risk Of Bias In Non-randomized Studies of Interventions.

Author (Year)	Country	Study Design	Sample Size	Population	Key Findings	Study Limitations	Primary Theme	Quality Appraisal Tool	Overall Risk of Bias
Kjøllesdal et al. (2020) [4]	Norway	Quantitative	NR	Immigrant patients	Lower satisfaction and delayed healthcare utilization	Self-reported data; cross-sectional design	Language barrier	ROBINS-I	Moderate
Krupic et al. (2016) [15]	Sweden	Qualitative	30	Immigrants	Interpreter-related language barriers	Small sample; qualitative design	Language barrier	CASP	Low
Amiri et al. (2015) [23]	Iran	Qualitative	18	Healthcare professionals	Insufficient transcultural competence	Small sample; subjective reporting	Transcultural incompetence	CASP	Low
Plaza Del Pino et al. (2013) [26]	Spain	Qualitative	12	Moroccan patients	Cultural misunderstanding and communication gaps	Small sample; cultural specificity	Transcultural incompetence	CASP	Low
Mangrio and Persson (2017) [31]	Sweden	Qualitative	14	Immigrant parents	Language barriers in child healthcare	Recall bias; small sample	Language barrier	CASP	Low
Aelbrecht et al. (2016) [29]	Belgium	Qualitative	22	Immigrant cancer patients	Poor communication and unmet informational needs	Disease-specific population	Language barrier	CASP	Low
Bachmann et al. (2014) [32]	Germany	Qualitative	15	Russian-speaking migrants	Miscommunication and mistrust	Limited representativeness	Language barrier	CASP	Low
Anoosheh et al. (2009) [36]	Iran	Qualitative	21	Nurses	Communication barriers causing stress	Self-reported data	Language barrier	CASP	Moderate
Biyikli Gültekin (2017) [37]	Austria	Quantitative	384	Turkish immigrants	Delayed access and dissatisfaction	Cross-sectional design	Language barrier	ROBINS-I	Moderate
Hunter-Adams and Rother (2017) [38]	South Africa	Qualitative	32	Immigrant patients	Language barriers impairing care quality	Context-specific findings	Language barrier	CASP	Low
Straiton and Myhre (2017) [40]	Norway	Qualitative	16	Immigrants	Navigational barriers in healthcare system	Small sample	Language barrier	CASP	Low
Smith and Ruston (2013) [41]	United Kingdom	Qualitative	20	Roma patients	Discrimination and healthcare avoidance	Self-reported experiences	Discrimination	CASP	Moderate
Ahrne et al. (2019) [42]	Sweden	Qualitative	24	Somali women	Perceived discrimination in antenatal care	Population-specific	Discrimination	CASP	Low
Reid and Taylor (2007) [43]	United Kingdom	Qualitative	19	Traveler women	Negative maternity care experiences	Small sample; cultural specificity	Discrimination	CASP	Moderate
Abdu et al. (2016) [44]	United Kingdom	Qualitative	17	South Asian families	Lack of culturally adapted counselling	Limited generalizability	Transcultural incompetence	CASP	Low

The included studies were conducted across diverse geographical regions, including Europe (Norway, Sweden, Spain, Belgium, Germany, Austria, and the United Kingdom), as well as Iran and South Africa, enhancing the contextual breadth and transferability of findings and providing limited but relevant perspectives from non-European settings (Table 2). Study populations included immigrant patients, healthcare professionals, and specific subgroups such as immigrant parents, migrant women, and ethnic minority families.

Across studies, three consistent thematic domains were identified. Language and communication barriers were frequently reported and were associated with miscommunication, delayed healthcare access, reduced satisfaction, and impaired quality of care [4,15,29,31,32,36-39,40]. Limited transcultural competence among healthcare professionals was identified as a contributing factor to communication challenges, professional frustration, and difficulties in delivering culturally responsive care [23,26,44]. Experiences of discrimination and differential treatment were also commonly described and were associated with mistrust, negative healthcare experiences, and avoidance of healthcare services [41-43].

Despite variations in study design, population characteristics, and healthcare settings, the consistency of findings across studies supports the robustness of the identified themes and suggests that barriers to healthcare access among immigrant populations are systemic rather than context-specific. Characteristics, key findings, limitations, thematic domain, and risk of bias of included studies are presented in Table 2.

Thematic analysis of the included studies identified three overarching themes: (i) transcultural competence, (ii) language deficiencies, and (iii) discrimination in healthcare. 

Transcultural Competence

Across the included studies, transcultural competence among healthcare professionals emerged as a central factor influencing the quality of care provided to immigrant patients. Healthcare professionals consistently emphasized the importance of knowledge in transcultural care, continuous education, and heightened awareness when caring for patients from diverse cultural backgrounds. Training focused on humanistic values, respect, empathy, patient rights, and effective communication was highlighted as essential for delivering high-quality care [4,23].

Several studies reported that insufficient transcultural competence contributed to professional frustration and feelings of inadequacy among healthcare staff when caring for immigrant patients [23]. Challenges were particularly pronounced when cultural differences intersected with language barriers, resulting in communication breakdowns and fragmented care processes. In such situations, care that failed to incorporate cultural understanding was often perceived as inadequate by patients [24]. To address these challenges, multiple studies advocated for structured and continuous training in transcultural care to enhance professionals’ ability to understand patients’ health beliefs and behaviors within a cultural context [25,26]. However, previous research indicates that formal education in transcultural care remains limited during professional training, leaving healthcare staff insufficiently prepared [27].

Language Deficiencies

Language barriers were consistently described as a major obstacle to effective healthcare delivery and were reported from both patient and healthcare professional perspectives. Studies identified two primary dimensions of this issue: communication challenges faced by healthcare staff and adverse consequences for patients with limited proficiency in the host-country language [28]. Immigrant patients frequently identified language barriers as a key impediment to receiving satisfactory healthcare [29,30], with poor bidirectional communication negatively affecting patient-provider interactions [29,31].

Consequences of inadequate communication included reduced self-confidence, increased fear and anxiety, and uncertainty regarding medical information, sometimes leading patients to guess the meaning of clinical instructions [31-33]. Inadequate understanding was associated with misdiagnosis, incorrect medication use, and premature discontinuation of treatment [30,34]. From the healthcare professionals’ perspective, communication difficulties led to avoidance of interactions with immigrant patients, contributing to anxiety, self-doubt, and concerns about providing suboptimal care [35].

Language barriers also increased workload and time demands for staff, exacerbating stress in already resource-constrained healthcare settings. Patients with limited language proficiency were more likely to receive incomplete information, struggle to follow treatment recommendations, make frequent emergency department visits, undergo more diagnostic testing, and report greater dissatisfaction with care [23,36-38]. Although interpreter services were introduced as a mitigating strategy, several studies indicated that these services were not always sufficient to fully address communication challenges [39,40]. Cultural factors further compounded language-related difficulties, including perceived lack of empathy, ignored patient questions, and mistrust toward healthcare professionals, ultimately contributing to delayed care-seeking and worsened health outcomes [15,37-39].

Discrimination in Healthcare

Discrimination against immigrant patients was reported across multiple studies and manifested in several forms, including prejudice, lower prioritization of care, and differential treatment. One study from England focusing on Roma patients described experiences of prejudice and discrimination that negatively affected psychological well-being, social withdrawal, and reluctance to seek healthcare [41]. Other studies similarly found that perceived prejudice increased vigilance and reduced healthcare utilization, particularly when patients encountered discriminatory or stigmatizing remarks from healthcare providers [42].

Discriminatory experiences also extended to perceptions of immigrant children being viewed as more disruptive in waiting rooms and negative attitudes toward family size among immigrant families [42,43]. In home healthcare settings, participants reported that standardized counseling approaches disregarded their cultural parenting practices, leaving them feeling misunderstood and undervalued [44]. Lower prioritization of care was frequently described, with participants perceiving that their ethnicity influenced the timeliness and quality of care received, particularly when compared with native-born or White patients [45]. Such experiences fostered fear of stigmatization and further discouraged healthcare-seeking behavior.

Experiences of special or differential treatment based on ethnicity, skin color, or religion were also reported. These included lack of attention, intrusive curiosity regarding bodily differences, and stereotyping of non-White patients. Although some participants minimized these encounters, such experiences contributed to feelings of alienation and reinforced perceptions of unequal treatment within healthcare systems [46,47].

Overall, the included studies demonstrated predominantly low to moderate risk of bias (Table 2). Methodological limitations were mainly related to participant selection and reporting transparency, which are common in qualitative and observational research designs. 

Discussion

Summary of Main Findings

This systematic review synthesized qualitative and quantitative evidence to identify the most common barriers encountered by immigrant patients when accessing healthcare services. Using Friberg’s five-step analytical framework [21], three interrelated themes consistently emerged: transcultural competence, language barriers, and discrimination in healthcare. The convergence of findings across geographically diverse studies strengthens the credibility and transferability of the results and suggests that these barriers are systemic rather than context-specific.

Interpretation in the Context of Existing Literature

Transcultural competence was consistently identified as a prerequisite for safe, respectful, and effective healthcare delivery. Healthcare professionals emphasized the importance of empathy, cultural awareness, and continuous education when caring for patients from diverse backgrounds [23-27]. Conversely, insufficient transcultural knowledge was associated with professional frustration, communication breakdowns, and reduced ability to provide culturally appropriate care [23-27]. Previous studies have similarly demonstrated that culturally responsive care improves patient engagement and healthcare effectiveness, although cultural differences may sometimes influence treatment acceptance [48]. Active patient participation and culturally sensitive dialogue were shown to facilitate individualized and context-adapted care planning [49].

Language barriers emerged as one of the most significant obstacles to effective healthcare delivery. Ineffective communication negatively affected patient-provider interactions, reduced patient confidence, and increased anxiety [28-32]. Healthcare professionals reported increased stress and fear of clinical errors when communication was inadequate, often relying on assumptions or incomplete information. Consequences included miscommunication, compromised care quality, increased emergency department utilization, higher diagnostic testing rates, and reduced patient satisfaction [33-37]. Delays in interpreter access, misunderstanding of medical information, and difficulties navigating healthcare systems were frequently reported [50,51]. Similar findings observed among internally displaced populations suggest that communication barriers reflect broader systemic challenges in healthcare access rather than issues unique to international migration [52].

Discrimination against immigrant patients was frequently described and manifested through prejudicial attitudes, differential treatment, and lower prioritization of care [42-45]. Patients reported feeling undervalued, stigmatized, or treated as less deserving of care. Discriminatory experiences, including stereotyping and differential attention based on ethnicity, religion, or cultural background, contributed to mistrust and reluctance to seek healthcare [46,47]. Such practices were associated with reduced trust in healthcare systems and potential adverse health outcomes [53], contradicting fundamental ethical and legal principles of equality and patient-centered care [54-56].

Implications for Clinical Practice, Policy, and Research

From a clinical perspective, integrating structured transcultural competence training into medical and nursing curricula, with measurable evaluation components, may improve culturally responsive care and strengthen patient-provider communication. Ensuring timely and reliable access to professional interpreter services is essential for safe clinical decision-making and reducing preventable errors. At the policy level, healthcare institutions should implement monitoring and accountability frameworks to identify and address discriminatory practices and promote equitable healthcare delivery.

Future research should focus on evaluating targeted interventions, including transcultural competence training, interpreter services, and anti-discrimination programs, with emphasis on measurable clinical outcomes, patient safety, and healthcare utilization. Longitudinal and comparative studies are needed to assess the impact of structural and policy-level interventions on healthcare equity and patient outcomes.

Strengths and Limitations

A major strength of this study is the systematic synthesis of both qualitative and quantitative research, allowing for a comprehensive understanding of immigrant patients’ experiences within healthcare systems. The inclusion of studies from multiple geographical regions enhances the transferability of the findings and suggests that the identified challenges are not confined to specific healthcare settings or countries.

The use of an established analytical framework (Friberg’s model) provided a structured and transparent approach to data synthesis, strengthening the methodological rigor of the analysis. Furthermore, adherence to PRISMA guidelines improved the transparency and reproducibility of the review process [21]. The identification of consistent themes across diverse contexts reinforces the credibility of the results and supports their relevance to international healthcare systems.

Several limitations should be considered when interpreting the findings of this review. First, although both qualitative and quantitative studies were included, the synthesis relied predominantly on qualitative evidence. While qualitative research provides rich contextual insight into patient experiences and healthcare interactions, it limits the ability to establish causal relationships, quantify effect sizes, or assess the magnitude of associations between identified barriers and clinical outcomes. Consequently, the findings should be interpreted as explanatory and hypothesis-generating rather than confirmatory.

Second, the inclusion of only English-language publications may have introduced language bias and led to the exclusion of relevant studies conducted in non-English-speaking settings. This limitation is particularly important, given that migration and healthcare access are global phenomena, and perspectives from regions where English is not the primary academic language may be underrepresented. As a result, the transferability of findings to certain cultural and healthcare contexts may be limited.

Third, substantial heterogeneity was observed across included studies with respect to study design, populations, healthcare systems, and outcome measures. Differences in immigration status, socioeconomic background, healthcare infrastructure, and policy environments may have influenced reported experiences and outcomes. This heterogeneity limits direct comparability between studies and precludes quantitative synthesis, potentially affecting the consistency and generalizability of conclusions.

Fourth, many included studies relied on self-reported patient or provider experiences, introducing potential recall bias, reporting bias, and subjective interpretation. Perceptions of discrimination, communication barriers, and cultural competence may vary depending on individual expectations, cultural norms, and prior healthcare experiences, which may influence the reliability and comparability of reported findings.

Fifth, variability in the conceptualization and measurement of key constructs, such as transcultural competence, language barriers, and discrimination, across studies may have affected thematic synthesis and interpretation. Differences in definitions, measurement tools, and analytical approaches may introduce interpretive variability and limit the precision of cross-study comparisons.

Sixth, potential publication bias cannot be excluded. Studies reporting negative or neutral findings, or those conducted in underrepresented populations or resource-limited settings, may be less likely to be published. This may result in overrepresentation of studies identifying barriers, potentially influencing the perceived consistency and strength of the findings.

Seventh, although methodological quality appraisal indicated an overall moderate risk of bias, variations in study quality, sample size, and methodological rigor across included studies may influence confidence in the findings. Some studies lacked detailed reporting on recruitment strategies, reflexivity, or data saturation, which may affect interpretive transparency.

Finally, most included studies were conducted in middle- to high-income healthcare settings, which may limit applicability to low-resource environments where structural barriers, workforce limitations, and access to interpreter services may differ substantially. Therefore, caution is warranted when generalizing these findings across diverse healthcare systems.

Collectively, these limitations may influence the strength, precision, and generalizability of the conclusions. While the consistency of themes across diverse contexts supports the robustness of the findings, they should be interpreted with appropriate caution, particularly when informing clinical practice, policy development, or implementation strategies.

Risk of Bias and Confidence in Findings

Although methodological variability and potential sources of bias were identified, the overall risk of bias across included studies was considered moderate and acceptable relative to the aims of this review. Potential biases include heterogeneity in outcome definitions, reliance on self-reported data, and language restriction. However, the consistency of findings across diverse study designs, populations, and healthcare contexts strengthens confidence in the robustness of the identified themes, supporting moderate-to-high confidence in the overall conclusions.

Equity and Relevance to Low-Resource Settings

The barriers identified in this review may be even more pronounced in low-resource and underserved healthcare settings, where access to interpreter services, transcultural training, and institutional equity frameworks may be limited. Structural constraints, workforce shortages, and limited healthcare infrastructure may exacerbate communication barriers and inequities in care delivery. Addressing these challenges requires scalable, context-sensitive strategies, including community-based communication support, culturally responsive care models, and policy frameworks promoting inclusive and equitable healthcare access. Strengthening equity-oriented healthcare delivery is essential to improving outcomes among migrant and other vulnerable populations globally.

## Conclusions

This systematic review demonstrates that immigrant patients face multiple, interconnected barriers when accessing healthcare services. Limited transcultural competence among healthcare professionals, persistent language barriers, and experiences of discrimination were consistently identified as key factors negatively affecting healthcare interactions, patient safety, satisfaction, and clinical outcomes, while also contributing to increased workload and professional strain among healthcare staff.

The consistency of findings across diverse healthcare systems indicates that these challenges are systemic. From a clinical perspective, improving culturally responsive care requires integrating structured transcultural competence training into medical and nursing education, ensuring reliable access to professional interpreter services, and implementing institutional measures to reduce discriminatory practices in healthcare delivery. Strengthening communication and cultural understanding is essential for improving patient safety, trust, and quality of care in increasingly diverse populations.

Future research should evaluate the clinical impact and effectiveness of targeted interventions, particularly transcultural competence training, interpreter services, and anti-discrimination strategies, on patient outcomes, healthcare quality, and equity. Addressing these barriers is fundamental to delivering safe, effective, and patient-centered care for immigrant populations.

## Appendices

Full Electronic Search Strategy - PubMed/MedLINE

(Immigrants OR "Immigrant populations" OR "Migrant" OR "Migrants" OR "Refugees" OR "Asylum seekers" OR "Foreign-born" OR "Ethnic minorities") AND ("Health services accessibility"[MeSH] OR "Healthcare access" OR "Health care utilization" OR "Access to care" OR "Barriers to healthcare" OR "Healthcare disparities") AND ("Communication barriers" OR "Language barriers" OR "Interpreter services" OR "Health communication") AND ("Cultural competence" OR "Transcultural care" OR "Cross-cultural care" OR "Culturally competent care") AND ("Discrimination" OR "Healthcare discrimination" OR "Inequality" OR "Health inequities")

Limits applied:

Humans

English language

Publication date: January 2005 - August 2023

The final search was executed on 31 August 2023.
